# The effect of *Toxoplasma gondii* infection on galectin-9 expression in decidual macrophages contributing to dysfunction of decidual NK cells during pregnancy

**DOI:** 10.1186/s13071-024-06379-2

**Published:** 2024-07-10

**Authors:** Xiao Wang, Shuyan Wang, Xiaoyan Xu, Yuzhu Jiang, Liqin Ren, Haixia Zhang, Zhidan Li, Xianbing Liu, Xuemei Hu, Yushan Ren

**Affiliations:** 1 Department of Immunology, Binzhou Medical University, Yantai, 264003 Shandong People’s Republic of China; 2 Department of Microbiology, Binzhou Medical University, Yantai, 264003 Shandong People’s Republic of China

**Keywords:** *Toxoplasma gondii*, Gal-9, dMφ, dNK, Adverse pregnancy outcomes

## Abstract

**Background:**

*Toxoplasma gondii* infection causes adverse pregnancy outcomes by affecting the expression of immunotolerant molecules in decidual immune cells. Galectin-9 (Gal-9) is widely expressed in decidual macrophages (dMφ) and is crucial for maintaining normal pregnancy by interacting with the immunomodulatory protein T-cell immunoglobulin and mucin domain-containing molecule 3 (Tim-3). However, the effects of *T. gondii* infection on Gal-9 expression in dMφ, and the impact of altered Gal-9 expression levels on the maternal–fetal tolerance function of decidual natural killer (dNK) cells, are still unknown.

**Methods:**

Pregnancy outcomes of *T. gondii*-infected C57BL/6 and *Lgals9*^−/−^ pregnant mice models were recorded. Expression of Gal-9, c-Jun N-terminal kinase (JNK), phosphorylated JNK (p-JNK), and Forkhead box protein O1 (FOXO1) was detected by western blotting, flow cytometry or immunofluorescence. The binding of FOXO1 to the promoter of *Lgals9* was determined by chromatin immunoprecipitation–polymerase chain reaction (ChIP-PCR). The expression of extracellular signal-regulated kinase (ERK), phosphorylated ERK (p-ERK), cAMP-response element binding protein (CREB), phosphorylated CREB (p-CREB), T-box expressed in T cells (T-bet), interleukin 10 (IL-10), and interferon gamma (IFN-γ) in dNK cells was assayed by western blotting.

**Results:**

*Toxoplasma gondii* infection increased the expression of p-JNK and FOXO1 in dMφ, resulting in a reduction in Gal-9 due to the elevated binding of FOXO1 with *Lgals9* promoter. Downregulation of Gal-9 enhanced the phosphorylation of ERK, inhibited the expression of p-CREB and IL-10, and promoted the expression of T-bet and IFN-γ in dNK cells. In the mice model, knockout of *Lgals9* aggravated adverse pregnancy outcomes caused by *T. gondii* infection during pregnancy.

**Conclusions:**

*Toxoplasma gondii* infection suppressed Gal-9 expression in dMφ by activating the JNK/FOXO1 signaling pathway, and reduction of Gal-9 contributed to dysfunction of dNK via Gal-9/Tim-3 interaction. This study provides new insights for the molecular mechanisms of the adverse pregnancy outcomes caused by* T. gondii.*

**Graphical Abstract:**

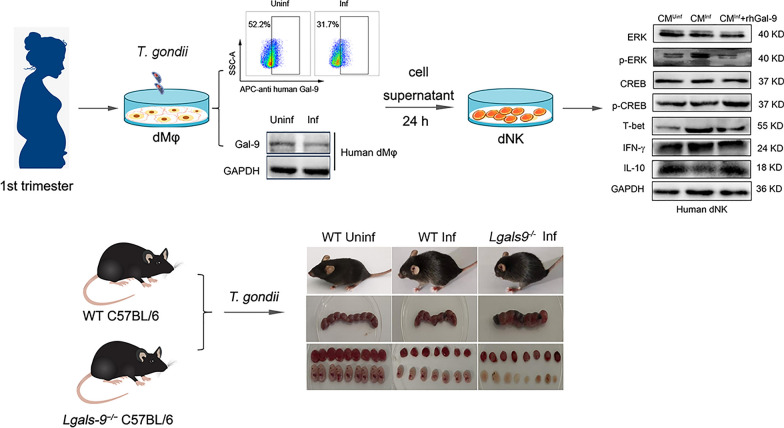

**Supplementary Information:**

The online version contains supplementary material available at 10.1186/s13071-024-06379-2.

## Background

*Toxoplasma gondii*, an intracellular parasite, is the causative pathogen of toxoplasmosis, which is a widespread zoonotic disease [1, 2]. *Toxoplasma gondii* is an opportunistic pathogenic parasite, especially in immunocompromised individuals. When pregnant women are infected with *T. gondii*, vertical transmission of this parasite results in congenital toxoplasmosis in newborns, including congenital defects and damage to nerves, eyes, or other organs [3]. The maternal–fetal interface comprises the maternal-derived decidua and the fetal-derived placenta [4]. Various immune cells are resident at the maternal–fetal interface and are important for the maintenance of normal pregnancy [5]. It is reported that decidual immune cells in early human pregnancy predominantly consist of natural killer (NK) cells (approximately 70%) and macrophages (approximately 20%), with variable proportions of T cells (approximately 10–20%) and scarce presence of dendritic cells (DC), B cells, and natural killer T (NKT) cells [6, 7]. These decidual immune cells, along with their cytokines, play important roles in maintaining normal pregnancy outcomes. Decidual macrophages (dMφ), as one of the main decidual immune cells, contribute to maintaining maternal–fetal tolerance, trophoblast invasion, and spiral artery remodeling during early pregnancy [8–10]. The gene expression profile of dMφ tends to be of the M2 phenotype during early pregnancy [11]. In humans, the dMφ express CD163, CD209, CD206, and DC-SIGN on their surfaces and predominantly secrete interleukin 10 (IL-10) [12]. DMφ also produces other immune tolerance factors such as indoleamine 2,3-dioxygenase (IDO) to sustain its biological function [13].

Galectin-9 (Gal-9) is involved in the modulation of the endometrial immune system, which is an essential member of the galectin family encoded by the *Lgals9* gene and expressed in the nucleus, cell membrane, and cytoplasm of cells [14, 15]. It is expressed by multiple cells at the maternal–fetal interface including trophoblasts, stromal cells, and dMφ. The biological functions of Gal-9 are related to immunomodulation, reproductive function maintenance, and facilitating establishment of pregnancy [16]. It is reported that, compared with non-pregnant women, plasma Gal-9 concentration is significantly elevated in normal pregnant women, with a high level of Gal-9 in the trophoblast cells of full-term placental tissues [17]. However, Gal-9 expression levels are reduced in trophoblast cells of villous tissue and in plasma in patients with miscarriage [18]. These findings highlight the crucial role of Gal-9 in maintaining maternal–fetal immune tolerance during normal pregnancy. As *T. gondii* infection is known to cause abortion by inhibiting the expression of immune negative molecules such as T-cell immunoglobulin domain and mucin domain-containing protein3 (Tim-3), LILRB4, and B7-H4 on various decidual immune cells [19–21], in this study we investigate the impact of *T. gondii* infection on Gal-9 expression in dMφ.

Forkhead box protein O1 (FOXO1) has multiple biological functions. It is reported that FOXO1 may suppress the transcription of *Lgals9* by binding to the promoter [22], and the bioactivity of FOXO1 can be manipulated by c-Jun N-terminal kinase (JNK) [23]. Moreover, the JNK signaling pathway can be efficiently activated during *T. gondii* infection [24]. Hence, it is important to explore whether *T. gondii* infection alters Gal-9 expression in dMφ through the JNK/FOXO1 signaling pathway.

At the maternal–fetal interface, decidual NK (dNK) cells represent the predominant leukocyte population, characterized by surface markers CD56^+^ and CD16^−^ in humans, and CD122^+^ and CD3^−^ in mice [25]. A significant portion of dNK cells express Tim-3, which is a receptor of Gal-9 and regulates dNK cell function at the maternal–fetal interface in early pregnancy [26]. However, whether Gal-9 from dMφ binds with Tim-3 to influence dNK cell function during *T. gondii* infection remains unclear. This study reveals a mechanism of reduced Gal-9 expression in dMφ following *T. gondii* infection, through the activation of the JNK/FOXO1 signaling pathway. Diminished Gal-9 levels in dMφ result in elevation of phosphorylated extracellular signal-regulated kinase (ERK) by binding to Tim-3, which further inhibits IL-10 expression and increases interferon gamma (IFN-γ) expression in dNK cells. This study also aims to elucidate the underlying molecular mechanisms whereby *T. gondii* infection causes dysfunction of dNK cells due to downregulation of Gal-9 in dMφ, which offers novel insights into the immune molecular pathways contributing to adverse pregnancy outcomes associated with *T. gondii* infection.

## Methods

### *Toxoplasma gondii* RH strain

*Toxoplasma gondii* RH tachyzoites were propagated and maintained in human foreskin fibroblast (HFF) cells cultured in Dulbecco’s modified Eagle medium (DMEM) medium supplemented with 10% fetal bovine serum (FBS) according to a previous study. After co-culturing HFF cells with *Toxoplasma* tachyzoites for 48 h, the cultures were collected. The cells were then removed by centrifugation at 5000×*g* for 5 min, and the tachyzoites remaining in the supernatant were purified by centrifugation at 4000×*g* for 7 min.

### Mice

C57BL/6 wild-type (WT) mice, 6 to 8 weeks old, were purchased from Ji’nan Pengyue Laboratory Animal Breeding Co., Ltd. (Jinan, China). *Lgals9*-knockout (*Lgals9*^*−/−*^) C57BL/6 mice were obtained from GemPharmatech Co., Ltd. (Nanjing, China). All mice were housed in the specific-pathogen-free (SPF) animal facility of Binzhou Medical University under controlled conditions, with temperature maintained at 23 ± 2 °C and a 12/12-h light/dark cycle. The experimental protocols were approved by the Animal Ethics Committee of Binzhou Medical University.

### Mouse experiments

For the *T. gondii*-infected pregnant mouse model, 8-week-old female C57BL/6 WT mice and *Lgals9*^*−/−*^ mice were mated with corresponding male mice at a ratio of 2:1. The appearance of the copulatory plug was designated as day 0 of gestation. In the infected group, pregnant mice were intraperitoneally infected with 300 *T. gondii* tachyzoites on gestation day (GD) 8. Pregnant mice in the control group were inoculated with phosphate-buffered saline (PBS).

### Cell preparation of mice

The uteri and placentas of mice were carefully separated and thoroughly washed with cold PBS. Subsequently, the tissues were finely minced into small fragments and enzymatically digested using 0.1% collagenase type IV (Sigma-Aldrich, MO, USA) and 25 IU/ml DNase I (Sigma-Aldrich) for 1 h at 37 °C with agitation. Following digestion, the tissue pieces were filtered through a 48-μm sterile mesh to obtain a cell suspension. Mononuclear cells were isolated from the white film layer after Ficoll density gradient centrifugation performed in mouse lymphocyte separation medium (TBD Science, Tianjin, China). Finally, the collected cells were suspended in cold PBS and utilized for subsequent flow cytometry analysis.

### Human sample collection

This study was approved by the Ethics Committee of Binzhou Medical University, Yantai, China, and was conducted in accordance with the Declaration of Helsinki. Decidual tissues were collected from the placentas of healthy pregnant women who had undergone termination of pregnancy between 6 and 8 weeks of gestation. The patients all read and signed informed consent forms.

### Isolation and purification of human dMφ and dNK

The decidual tissues were washed with cold PBS, cut into small fragments using ophthalmic scissors, and digested with 0.1% collagenase IV (Sigma-Aldrich) and 25 IU/ml DNase I (Sigma-Aldrich) in an incubator at 37 °C for 60 min. The resulting suspension was filtered through 48-µm mesh and washed twice in cold PBS. The decidual mononuclear cells were isolated and purified by Ficoll density gradient centrifugation. These cells were then employed for flow cytometry analysis. For further purification, the dMφ cells were isolated using a human CD14-positive isolation kit (STEMCELL Technologies, Toronto, Canada) according to the manufacturer’s instructions. The dNK cells were purified using a human NK cell isolation kit (STEMCELL Technologies) according to the manufacturer’s instructions. The purified dMφ cells were infected with *T. gondii* tachyzoites and analyzed using western blotting, immunofluorescence, and chromatin immunoprecipitation (ChIP)–polymerase chain reaction (PCR) methods. The purified dNK cells were used for subsequent co-culture experiments. The specific culture and treatment conditions of the purified dMφ and dNK are described for each experiment.

### Human decidual mononuclear cell culture and treatment

The mononuclear cells were evenly divided into two groups: the uninfected group and the infected group. *Toxoplasma gondii* tachyzoites were added to the cells of the infected group at a ratio of 1:3 (*T. gondii* tachyzoites/cells). All cells were cultured in RPMI-1640 medium supplemented with 10% FBS (Gibco, MA, USA), 100 IU/ml streptomycin, and 100 IU/ml penicillin for 24 h at 37 °C in a humidified 5% CO_2_ incubator. Subsequently, all cells were prepared for flow cytometry staining and analysis.

### Flow cytometry staining and analysis

The prepared human and murine decidual mononuclear cell (1 × 10^6^ cells of each group) suspensions were first stained with antibodies against membrane molecules and then with antibody of intracellular molecule Gal-9, after the cells were treated with intracellular fixation and permeabilization buffer set according to the manufacturer’s instructions (eBioscience, CA, USA). The experiments were carried out at least three times. Data were analyzed using a BD FACSCanto™ II flow cytometer (BD Biosciences, NJ, USA) and FlowJo analysis software (version 10.0, FlowJo LLC, OR, USA).

### M2 macrophage differentiation of THP-1 cells

THP-1 cells were added to 100-mm cell culture dishes (8 × 10^6^ cells/dish) and supplemented with 50 nM phorbol 12-myristate 13-acetate (PMA) to induce differentiation into macrophages. After 24 h, the supernatant was discarded and fresh medium was added, supplemented with 0.1 μM medroxyprogesterone acetate (MPA). After 72 h, the cells were polarized into M2 macrophages. Flow cytometry was used to detect the phenotype of M2 macrophages. Antibodies against CD14, CD206, and CD209 were used for surface staining according to the manufacturer’s instructions. The induced THP-1 cells were transfected with pcDNA3.1-FOXO1 using jetPRIME^®^ reagent (Polyplus, Illkirch, France), followed by *T. gondii* infection; then the cells were harvested and used for detecting Gal-9 by western blotting.

### Western blotting analysis

Purified human dMφ cells were infected with *T. gondii* tachyzoites at a ratio of 1:3 (*T. gondii* tachyzoites/cells) with or without 10 μM SP600125 (JNK inhibitor [JNKi]), 10 μg/ml AS1842856 (FOXO1 inhibitor [FOXO1i]), and 10 μg FOXO1 overexpression plasmid. The supernatant of *T. gondii*-infected dMφ cells was collected and added into the purified dNK cells, supplemented with 10 μg of recombinant human Gal-9 protein (rhGal-9), 10 μg/ml Tim-3 neutralizing antibody (α-Tim-3), and 10 μM PD98059 (p-ERK inhibitor [p-ERKi]). The dMφ and dNK cells were washed with PBS and lysed using RIPA cell lysis buffer. The total protein concentration was determined using a BCA kit (Solarbio, Beijing, China). Subsequently, 30 μg of protein was separated by sodium dodecyl sulfate–polyacrylamide gel electrophoresis (SDS-PAGE) using a 12% Bis–Tris gel (Solarbio) and then transferred onto a polyvinylidene fluoride (PVDF) membrane (Millipore, MA, USA). After blocking with 5% nonfat milk, the blots were probed with primary antibodies, including rabbit anti-Gal-9 (1:2000, Proteintech, Wuhan, China), rabbit anti-JNK, rabbit anti-phosphorylated JNK (p-JNK, 1:2000, Proteintech), rabbit anti-FOXO1 (1:2000, Proteintech), rabbit anti-ERK (1:2000, Proteintech), rabbit anti-phosphorylated ERK (p-ERK, 1:2000, Proteintech), rabbit anti-CREB [cyclic AMP (cAMP)-response element-binding protein] (1:2000, Proteintech), rabbit anti-phosphorylated CREB (p-CREB, 1:2000, Proteintech), rabbit anti-IL-10 (1:2000, Wanleibio, Shenyang, China), rabbit anti-T-bet [T-box expressed in T cells] (1:2000, Wanleibio), rabbit anti-IFN-γ (1:2000, Bioss, Beijing, China), and rabbit anti-GAPDH antibodies (1:40000, Proteintech). Subsequently, the PVDF membranes were incubated with horseradish peroxidase (HRP)-conjugated secondary antibodies (1:5000, Proteintech). The images were captured using the Bio-Rad ChemiDoc XRS+ imaging system (BioRad, CA, USA).

### Immunofluorescence analysis

The purified dMφ cells were cultured in 60-mm cell culture dishes, divided into an uninfected group and an RH strain-infected group. In the RH strain infection group, *T. gondii* parasites were added at a ratio of 1:3. After 24 h, all cells were fixed in 4% paraformaldehyde (PFA) at room temperature (RT) for 20 min, followed by cell permeabilization with 0.2% Triton X-100 for 10 min. After blocking with normal goat serum for 1 h at RT, the samples were then incubated with rabbit anti-Gal-9 antibody (Proteintech, 1:100 dilution) and mouse anti-FOXO1 antibody (Proteintech, 1:100 dilution) overnight in a humid chamber at 4 °C followed by Alexa Fluor 549- or 488-conjugated immunoglobulin G (IgG) incubation. After PBS washing, 4′,6-diamidino-2-phenylindole (DAPI, Sigma) was used to stain nuclei. Fluorescence signals were analyzed using an inverted fluorescence microscope (Olympus, Tokyo, Janpan). To detect the expression of Gal-9 in mouse placental tissue, placentas were prepared into frozen sections and stained with rabbit anti-Gal-9 antibody and Alexa Fluor 488-conjugated IgG.

### ChIP-PCR assay

ChIP assays were conducted using the Pierce Magnetic ChIP Kit (Thermo Fisher, MA, USA). The dMφ cells were fixed with 1% formaldehyde for 10 min at RT, with the fixation reaction quenched using glycine. Nuclei were isolated via cell lysis, followed by digestion using micrococcal nuclease (MNase) and sonication to achieve a fragment length of approximately 150–900 base pairs (bp). After purification, 5 μg of chromatin was utilized for each immunoprecipitation. For each immunoprecipitation, 5 μg of anti-FOXO1 antibody or control IgG was added to the digested, cross-linked chromatin. After eluting chromatin from antibody/protein G magnetic beads, the immunoprecipitated chromatin was purified and subjected to quantitative real-time PCR detection using specific primers. The primers used for ChIP-qPCR were synthesized by Sangon Biotech (Shanghai, China) and are listed in Additional file 1: Table S1.

### Reagents and antibodies

The reagents and antibodies used in the study are listed in Additional file 2: Table S2.

### Statistical analysis

Statistical analysis was conducted using GraphPad Prism 9.0 software (GraphPad Software, CA, USA). Data are presented as the mean ± standard error (SE). Unpaired and paired *t*-tests were utilized to identify differences. One-way analysis of variance (ANOVA) was applied with 95% confidence intervals. Significance was defined as *P* < 0.05.

## Results

### Abnormal pregnancy outcomes were associated with Gal-9 in *T. gondii*-infected mice

**Fig. 1:**
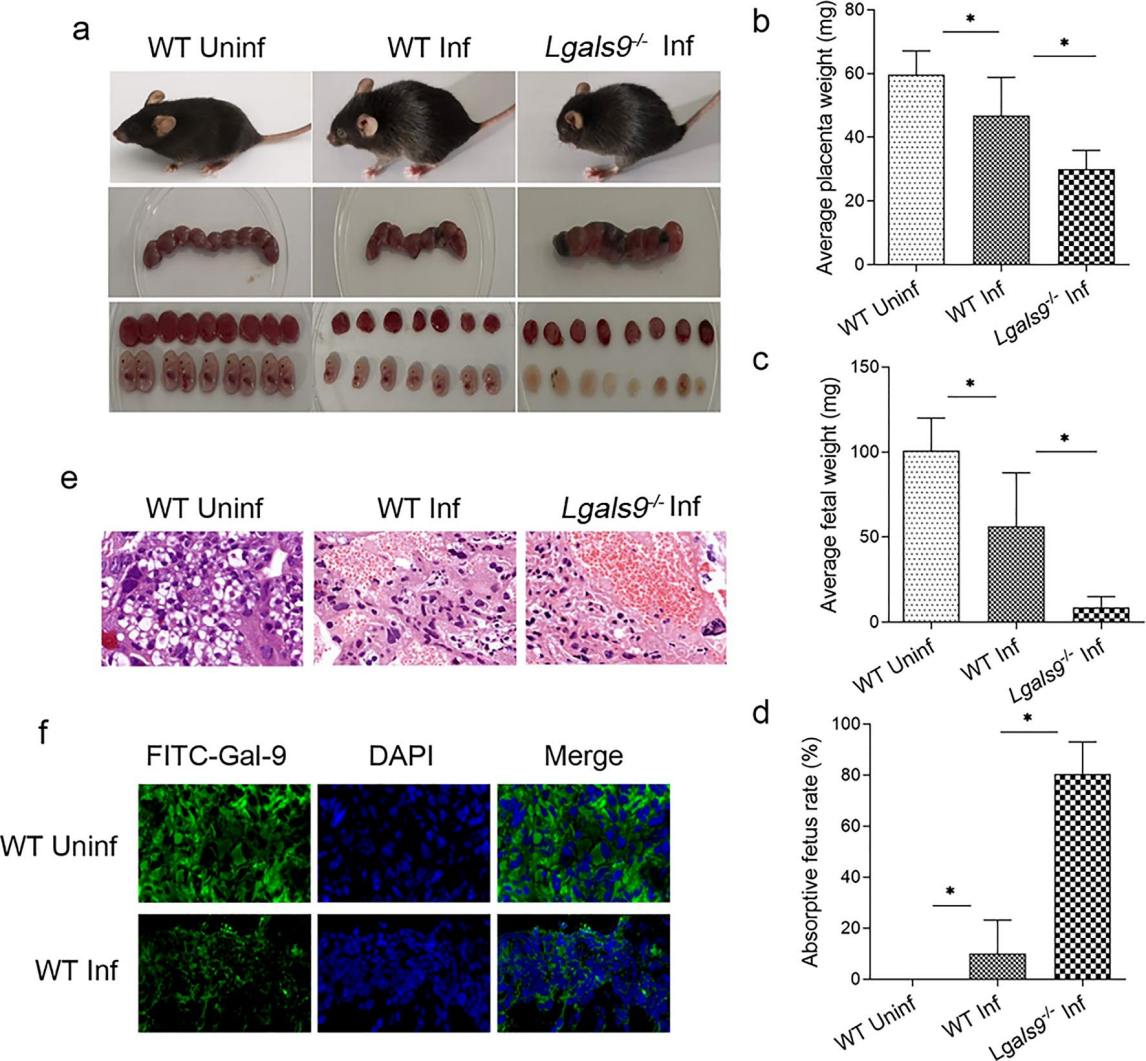
Abnormal pregnancy outcomes were associated with Gal-9 in *T. gondii*-infected mice. **a** Pregnancy outcomes of *T. gondii*-infected wild-type (WT) mice compared with *Lgals9* knockout (*Lgals9*^−/−^) mice. **b**–**d** Statistical analysis of placental weight, fetal weight, and fetal absorption rate in the uninfected WT group, infected WT group, and *Lgals9* knockout mice infected group. **e** Histopathological analysis of placental pathology in the uninfected WT group, infected WT group, and *Lgals9*^−/−^ mice. **f** Immunofluorescence was employed to detect Gal-9 expression in frozen sections of mouse placenta. The statistical analysis is presented as mean ± standard error (SE), with at least six pregnant mice in each group, and unpaired *t*-tests were performed (*P < 0.05, **P < 0.01)

To investigate the impact of Gal-9 on the adverse pregnancy outcomes caused by *T. gondii*, both WT and *Lgals9*^*−/−*^ (Gal-9 knockout) mice models infected with *T. gondii* were established, and the pregnancy outcomes were evaluated. For the *T. gondii*-infected pregnant mouse model, 8-week-old female C57BL/6 WT mice and *Lgals9*^*−/−*^ mice were mated with corresponding male mice at a ratio of 2:1. The appearance of the copulatory plug was designated as day 0 of gestation. In the infected group, pregnant mice were intraperitoneally infected with 300 *T. gondii* tachyzoites on GD 8. Pregnant mice in the control group were inoculated with PBS. Pregnancy outcomes were observed on GD 14. The experimental results indicated that adverse pregnancy outcomes in *Lgals9*^*−/−*^ mice were significantly more severe than those in WT mice under infection conditions (Fig. 1a). The placental weight of *Lgals9*^*−/−*^ mice was noticeably reduced compared to WT mice (Fig. 1b), along with a significant decrease in fetal weight (Fig. 1c). *Lgals9*^*−/−*^ mice exhibited a higher rate of fetal resorption (Fig. 1d). Hematoxylin and eosin (HE) staining revealed that, following *T. gondii* infection, *Lgals9*^*−/−*^ mice exhibited more pronounced disruption of placental tissue integrity, increased infiltration of inflammatory cells, and severe hemorrhaging compared to WT mice (Fig. 1e). Immunofluorescence was employed to detect the expression of Gal-9 in frozen sections of mouse placenta. The results revealed a significant decrease in Gal-9 expression in the placenta of the infected group compared with the uninfected group (Fig. 1f). These results indicated that *Lgals9*^*−/−*^ mice experienced more severe pregnancy outcomes following *T. gondii* infection, suggesting that *Lgals9* deficiency exacerbated adverse pregnancy outcomes.

### Gal-9 was decreased in dMφ after *T. gondii* infection

**Fig. 2:**
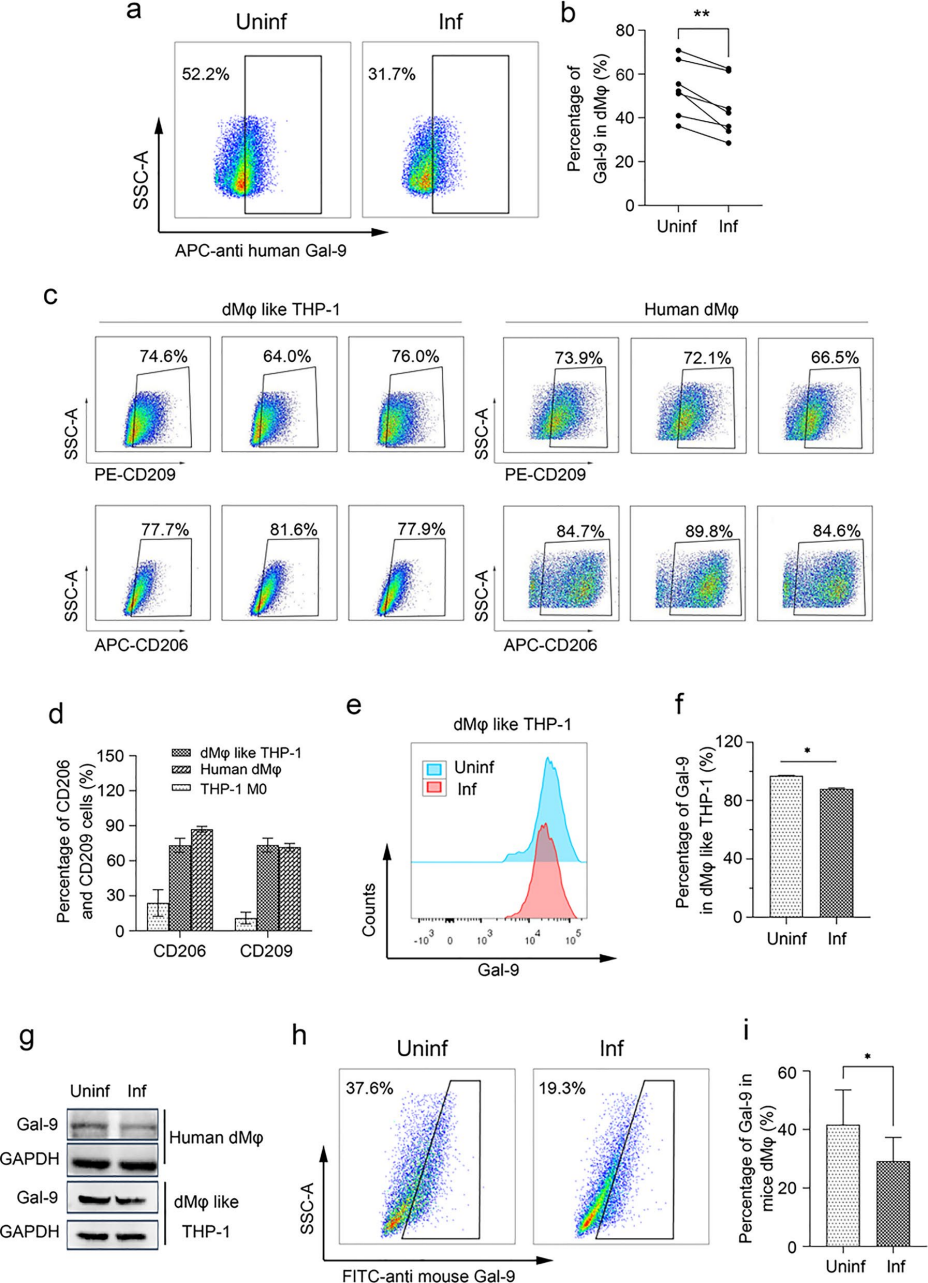
Gal-9 was decreased in dMφ after *T. gondii* infection. Flow cytometry was employed to evaluate the expression of Ga-9 in human dMφ (**a**, **b**). The expression of CD206 and CD209 was detected in dMφ-like THP-1 cells (**c**, **d**). Flow cytometry was used to evaluate the expression of Gal-9 in the dMφ-like THP-1 cell line (**e**, **f**). **g** Western blotting was conducted to assess the changes in expression of Gal-9 in human dMφ and dMφ-like THP-1 cell line after infection. **h**, **i** Flow cytometry was used to evaluate the expression of Gal-9 in mouse dMφ from infected and uninfected groups. The statistical analysis presented above displays mean ± SE. Each group in the in vivo experiments consisted of six pregnant mice and was subjected to the unpaired *t*-test, while each group in the in vitro experiments comprised at least three human decidual tissue specimens and underwent paired *t*-test (*P < 0.05, **P < 0.01)

To explore the impact of *T. gondii* infection on Gal-9 expression in various immune cells, we isolated human decidual immune cells at the maternal–fetal interface during early pregnancy. The expression of Gal-9 was found to be significantly decreased in dMφ infected with *T. gondii* (Fig. 2a, b). Simultaneously, we utilized flow cytometry to assess the expression of Gal-9 in THP-1-derived M2-type macrophages (dMφ-like THP-1). Firstly, the phenotype of dMφ-like THP-1 was assayed by staining CD206 and CD209 through flow cytometry detection. The expression of CD206 and CD209 was high in dMφ-like THP-1 cells as well as in human dMφ (Fig. 2c, d), which suggested that dMφ-like THP-1 could be used to investigate the mechanism in the subsequent experiments. We then found that Gal-9 expression was also reduced in these dMφ-like THP-1 cells after *T. gondii* infection (Fig. 2e, f). The western blotting results also indicated that *T. gondii* infection caused a decrease in Gal-9 expression in dMφ (Fig. 2g). Additionally, in vivo, *T. gondii* infection resulted in a remarkable reduction in Gal-9 in the dMφ of WT mice (Fig. 2h, i). These data collectively confirmed that *T. gondii* infection contributed to a significant decrease in Gal-9 expression within dMφ.

### *Toxoplasma gondii* infection attenuated Gal-9 expression in dMφ via the JNK/FOXO1 signaling pathway

**Fig. 3:**
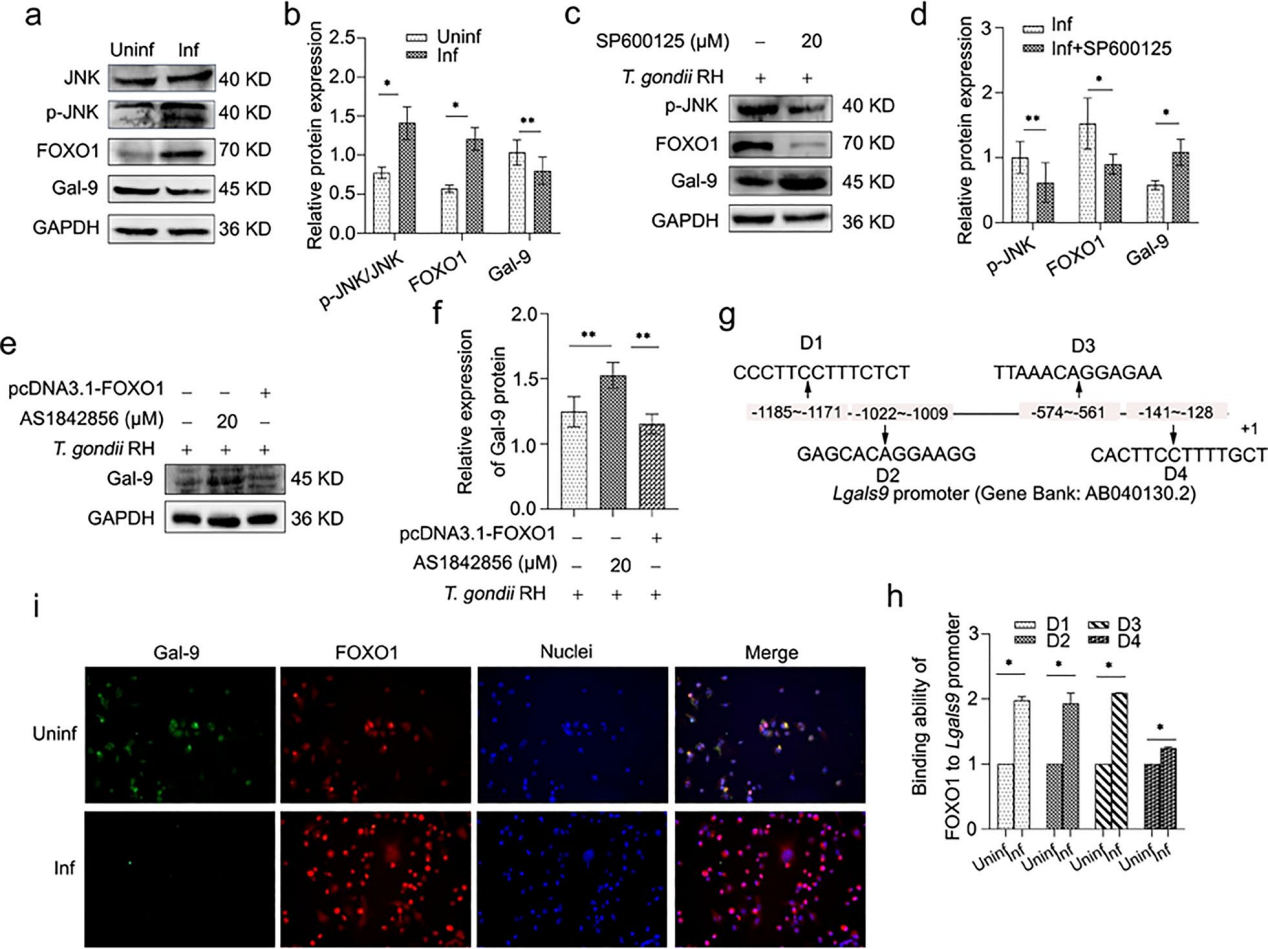
*Toxoplasma gondii* infection attenuated Gal-9 expression in dMφ via the JNK/FOXO1 signaling pathway. **a**, **b** Western blotting analysis was performed to evaluate the expression levels of p-JNK, FOXO1, and Gal-9 within human dMφ infected with *T. gondii* or not. **c**, **d** The expression levels of p-JNK, FOXO1, and Gal-9 were determined in infected dMφ treated with a JNK inhibitor. **e**, **f** Western blotting analysis was conducted to examine the expression levels of Gal-9 in infected human dMφ treated with a FOXO1 inhibitor or transfected with FOXO1 overexpression plasmid. **g** The schematic diagram illustrates the potential binding regions of FOXO1 with the promoter of *Lgals9*. **h** ChIP-qPCR analysis was used to detect the binding ability of FOXO1 to *Lgals9* promoter after infection. **i** Immunofluorescence analysis was performed to detect the expression of FOXO1 and Gal-9 in uninfected or infected human dMφ. The statistical analysis presented above is displayed as mean ± SE. For in vitro experiments, each group comprised at least three human decidual tissue specimens, and paired *t*-tests were conducted (*P < 0.05, **P < 0.01)

Previous studies have reported that FOXO1 acts as a transcriptional repressor to inhibit Gal-9 expression [22]. To elucidate the underlying mechanism of the impact of *T. gondii* infection on Gal-9 expression, western blotting analysis was performed to assess the expression levels of p-JNK and FOXO1 in dMφ infected with *T. gondii*. The results revealed an increase in both p-JNK and FOXO1 levels following *T. gondii* infection, accompanied by a decrease in Gal-9 expression (Fig. 3a, b). To investigate whether the expression level of Gal-9 in dMφ was regulated by JNK/FOXO1 during *T. gondii* infection in vitro, SP600125, a JNK inhibitor, was introduced to suppress phosphorylation of JNK in human dMφ. Additionally, a FOXO1 inhibitor, AS1842856, was used to inhibit FOXO1, while a FOXO1 recombined plasmid pcDNA3.1-FOXO1 was transfected into dMφ to upregulate FOXO1 expression levels in dMφ-like THP-1. Then western blotting assay was performed to analyze the expression of JNK, p-JNK, FOXO1, and Gal-9. The results suggested that SP600125 led to a decrease in the expression levels of p-JNK and FOXO1 in human dMφ or dMφ-like THP-1, but there was a significant increase observed in Gal-9 expression (Fig. 3c, d). Inhibition of FOXO1 by AS1842856 provoked an increase in Gal-9 expression, whereas overexpression of FOXO1 caused a decrease in Gal-9 expression (Fig. 3e, f). To determine the mechanism by which FOXO1 regulates Gal-9 expression, ChIP-qPCR assay was performed in *T. gondii*-infected dMφ cells. Primers targeting the Gal-9 promoter and anti-FOXO1 antibody were used for ChIP-qPCR. The results demonstrated that FOXO1 could directly bind to the promoter region of *Lgals9* (−1185 to −1171 bp, −1009 to −1022 bp, −574 to −561 bp, −141 to −128 bp), and this binding ability was enhanced following *T. gondii* infection (Fig. 3g, h). Immunofluorescence analysis similarly demonstrated an upregulation of FOXO1 expression and a downregulation of Gal-9 expression in *T. gondii*-infected dMφ cells (Fig. 3i). These findings suggest that the expression levels of Gal-9 following *T. gondii* infection were negatively regulated by the JNK/FOXO1 signaling pathway.

### The reduction in Gal-9 derived from dMφ promoted phosphorylation of ERK via the Gal-9/Tim-3 interaction, consequently impacting the function of dNK cells

**Fig. 4:**
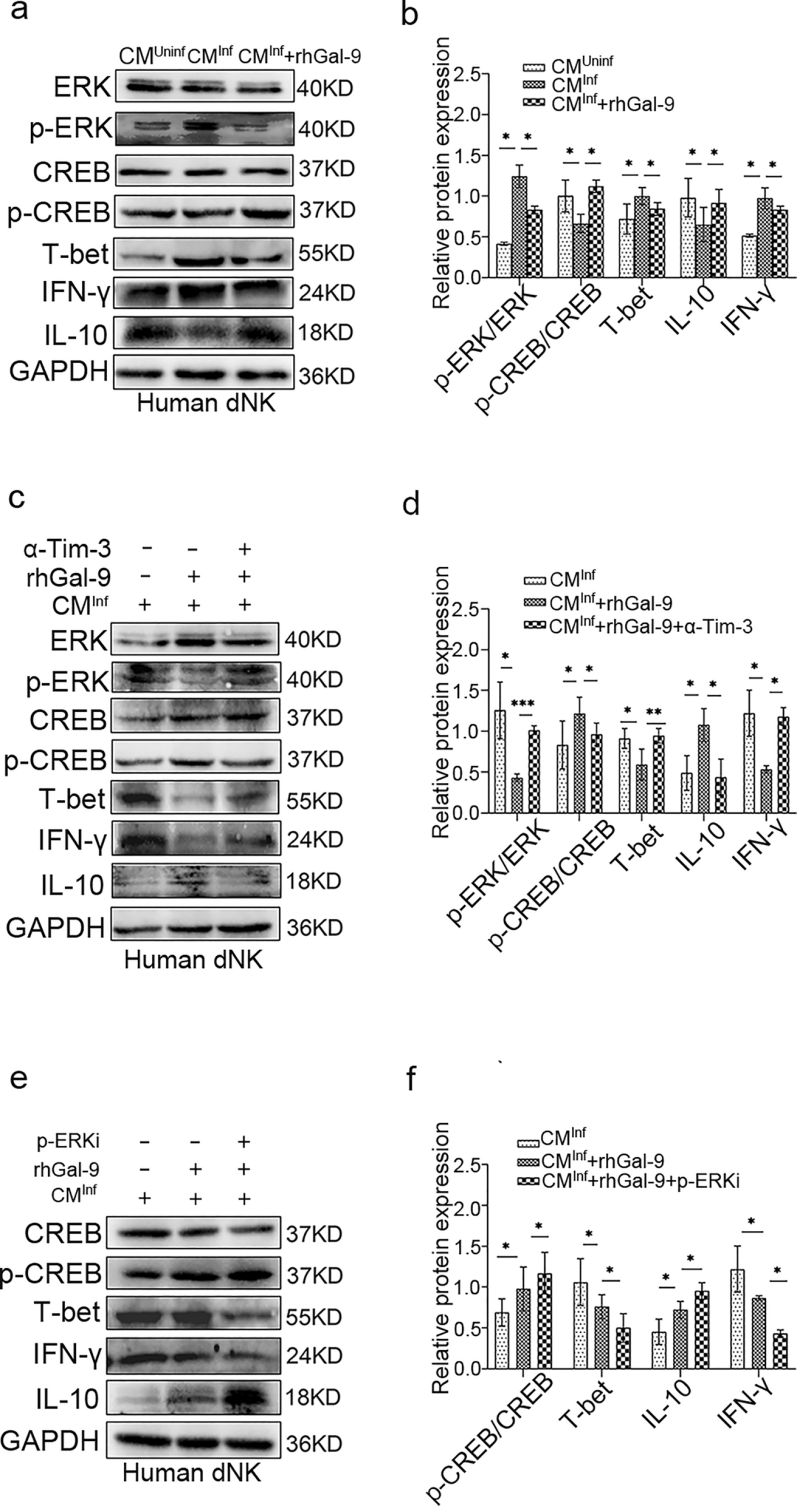
The reduction in Gal-9 derived from dMφ activated phosphorylation of ERK via the Gal-9/Tim-3 interaction, consequently impacting the function of human dNK cells. **a** The expression levels of p-ERK, p-CREB, T-bet, IL-10, and IFN-γ were detected using western blotting in dNK cells co-cultured with CM^Uninf^ and CM^Inf^ in the presence or absence of rhGal-9. **b** Statistical analysis was then conducted to analyze the results. **c** The expression levels of p-ERK, p-CREB, T-bet, IL-10, and IFN-γ in dNK cells were detected using western blotting in three groups: CM^Inf^, CM^Inf^ + rhGal-9, and CM^Inf^ + rhGal-9 + α-Tim-3. **d** Statistical analysis was then conducted to analyze the results. **e** The expression levels of p-CREB, T-bet, IL-10, and IFN-γ in dNK cells were detected using western blotting in three groups: CM^Inf^, CM^Inf^ + rhGal-9, and CM.^Inf^ + rhGal-9 + p-ERKi. **f** Statistical analysis was then conducted to analyze the results. The statistical analysis presented above is displayed as mean ± SE. For in vitro experiments, each group comprised at least three human decidual tissue specimens (*P < 0.05, **P < 0.01)

To elucidate the immune function of human dMφ following *T. gondii* infection, we prepared a conditioned medium (CM) containing secreted products from dMφ. To assess the impact of Gal-9 derived from dMφ on maternal immunity, we treated dNK cells with CM from human dMφ infected or not infected with *T. gondii*. We observed that CM from *T. gondii*-infected dMφ (CM^Inf^) significantly suppressed IL-10 expression in dNK cells while enhancing IFN-γ expression compared with CM from uninfected dMφ (CM^Uninf^) (Fig. 4a, b). To validate whether the reduction of Gal-9 affects human dNK cell function, we added rhGal-9 protein into the CM^Inf^. Following treatment with rhGal-9, we observed an increase in IL-10 expression and a decrease in IFN-γ expression in dNK (Fig. 4a, b). We also found that p-CREB and T-bet, downstream molecules of the ERK signaling pathway, were decreased and increased in dNK cells, respectively, under CM^inf^ co-culturing. Conversely, the addition of rhGal-9 efficiently promoted phosphorylation of CREB and suppressed the expression of T-bet (Fig. 4a, b). It is reported that p-CREB is a transcription factor of IL-10, and T-bet is a transcription factor of IFN-γ [27, 28].

Tim-3 has been reported to serve as a receptor for Gal-9, regulating immune cell functions. To validate whether Gal-9 secreted by dMφ affects the function of dNK cells by interacting with Tim-3 on dNK cells, we utilized a Tim-3 neutralizing antibody (α-Tim-3) to disturb their binding. The results revealed an increase in p-ERK expression, a decrease in IL-10 expression along with its transcription factor p-CREB, and an increase in expression of IFN-γ and T-bet, which is a classical transcriptional factor of IFN-γ, when α-Tim-3 was added into CM^inf^ (Fig. 4c, d). To further confirm whether the Gal-9/Tim-3 signaling pathway impacts dNK cell function by affecting ERK phosphorylation, PD98059 (a p-ERK inhibitor) was employed to suppress phosphorylation of ERK. The results showed that inhibition of ERK phosphorylation increased the expression of IL-10 and the phosphorylation of CREB, but decreased the expression of IFN-γ and T-bet (Fig. 4e, f). These results suggest that the reduction of Gal-9 in *T. gondii*-infected dMφ may impact the function of dNK cells by enhancing ERK phosphorylation via suppression of the interaction of Gal-9 and Tim-3.

## Discussion

Decidual macrophages, characterized by M2-like macrophages, are integral components of decidual immune cells at the maternal–fetal interface, crucial for maintaining maternal–fetal tolerance and trophoblast invasion [29]. The removal of macrophages in pregnant rats results in abortions, highlighting the significance of macrophages in sustaining normal pregnancy [16, 30]. Under normal pregnancy conditions, dMφ exhibits a high expression of immunosuppressive molecules such as IL-10, IDO, Tim-3 and LILRB4, contributing to their immunological tolerance effect [31–33]. However, in pregnancy complications such as pre-eclampsia (PE), premature birth and recurrent pregnancy loss (RPL), and miscarriages, dMφ displays an abnormal polarization, with high expression of inflammatory factors [32, 34]. *Toxoplasma gondii*, the pathogen associated with TORCH [toxoplasmosis, rubella, cytomegalovirus, herpes] syndrome, contributes to adverse pregnancy outcomes including preterm labor, miscarriage, birth defects, or stillbirth. A previous study reported a reduction in IL-10, Tim-3, or LILRB4 expression in dMφ in a mouse model of *T. gondii*-induced miscarriage [31, 33]. Nevertheless, Gal-9 is an important ligand of Tim-3, and there are no reports about whether *T. gondii* infection affects the expression level of the Gal-9 in dMφ.

Our investigation found that Gal-9 levels were significantly decreased in decidual tissue of mice following *T. gondii* infection, accompanied by serious adverse pregnancy outcomes in mice lacking Gal-9. In particular, *T. gondii* infection resulted in reduced placental and fetal mice weight in *Lgals9* knockout (*Lgals9*^−/−^) pregnant mice compared with WT pregnant mice. Flow cytometry analysis showed lower Gal-9 expression in dMφ of *T. gondii*-infected pregnant mice compared with uninfected pregnant mice. Similarly, in vitro, flow cytometry results revealed a significant reduction in Gal-9 expression in human primary dMφ or dMφ-like THP-1 following *T. gondii* infection, while Gal-9 levels in other immune cells such as dNK and decidual myeloid-derived suppressor cells (MDSC) remained unaffected after infection (data not shown). Additionally, western blotting assay revealed a significant decline in Gal-9 expression after infection in human dMφ. These results suggest that Gal-9 expression was reduced due to *T. gondii* infection, indicating its important role in abnormal pregnancy outcomes induced by *T. gondii* infection.

However, the underlying molecular mechanisms by which *T. gondii* infection reduced Gal-9 expression remain unclear. Previous studies have indicated that the upstream transcriptional activity of *Lgals9* can be downregulated by FOXO1 [22]. Additionally, it is reported that *T. gondii* infection is involved in increased phosphorylation of JNK, which induces an inflammatory response [24]. Moreover, JNK phosphorylation in turn induces activity of FOXO1, which mediates multiple signaling pathways [35]. Our experiments demonstrated a significant elevation in phosphorylation of JNK and expression of FOXO1 in human dMφ following *T. gondii* infection in vitro, suggesting a potential relationship between *T. gondii* infection and increased p-JNK and FOXO1 levels. However, further studies are needed to verify whether *T. gondii* affects the expression of Gal-9 by promoting JNK phosphorylation and activating FOXO1 in dMφ. To explore whether the decrease in Gal-9, which is caused by *T. gondii* infection in human dMφ, was regulated by activation of the JNK/FOXO1 signaling pathway, a JNK inhibitor, SP600125, was added to inhibit phosphorylation of JNK in the presence of *T. gondii* infection. The expression levels of FOXO1 and Gal-9 were detected using western blotting assay in the infection group with or without JNK inhibitor. The results showed that the JNK inhibitor decreased FOXO1 levels and increased Gal-9 levels following *T. gondii* infection, suggesting that in the presence of *T. gondii,* JNK phosphorylation affected the expression of FOXO1 and Gal-9 in dMφ. Furthermore, *T. gondii*-infected dMφ was treated with FOXO1 inhibitor or transfected with a plasmid pcDNA3.1-FOXO1, and the expression levels of Gal-9 were detected using western blotting assay, wherein the expression level of Gal-9 was increased in the infected dMφ treated with FOXO1 inhibitor and decreased in the infected dMφ with FOXO1 overexpression, suggesting that *T. gondii* infection inhibited the expression of Gal-9 through the activation of FOXO1. To examine whether *T. gondii* affects the binding of FOXO1 to *Lgals9* promoter, we conducted ChIP-qPCR using the dMφ-like THP-1 cell line, which was divided into uninfected and infected groups. The results revealed that binding of FOXO1 to the upstream promoter of *Lgals9* was enhanced post-infection, suggesting that FOXO1 could directly bind to the Gal-9 promoter and suppress its transcriptional expression following *T. gondii* infection.

Interleukin 10, crucial for maintaining normal maternal–fetal tolerance, is produced by dNK cells and dMφ in decidual immune cells [36, 37]. Our findings suggest that decreased Gal-9 expression in dMφ due to *T. gondii* infection further influenced the function of dNK cells by binding to Tim-3. This is evidenced by reduced IL-10 expression and increased IFN-γ expression in dNK cells. It is reported that Tim-3, a receptor for Gal-9, is downregulated after *T. gondii* infection, increasing IFN-γ expression through the Fyn/ERK signaling pathway, enhancing dNK cytotoxicity, and disrupting the balance of maternal–fetal tolerance [38, 39]. Activation of Tim-3 by Gal-9 inhibits the ERK signaling pathway and promotes IL-10 production [40, 41]. To investigate whether *T. gondii-*induced reduction of Gal-9 in dMφ affected the function of dNK cell by binding to Tim-3 and enhanced ERK phosphorylation, we collected conditioned medium (CM) of uninfected or infected dMφ in vitro. Co-culture of infected (CM^Inf^) and uninfected CM (CM^Uninf^) with purified human dNK cells, along with the addition of rhGal-9 protein, Tim-3 neutralizing antibody, or p-ERK inhibitor, revealed that phosphorylation of ERK was increased in co-cultured dNK cells treated with CM^Inf^ compared with CM^Uninf^, accompanied by decrease in p-CREB and IL-10 and an increase in T-bet and IFN-γ. p-CREB and T-bet are classic transcription factors of IL-10 and IFN-γ, respectively [27, 28]. RhGal-9 protein treatment increased the expression of p-CREB and IL-10 and decreased the expression of T-bet and IFN-γ, which could be reversed by adding Tim3 neutralizing antibody, indicating that Gal-9 can affect the function of dNK by Tim-3. CM^Inf^ co-cultured dNK cells treated with rhGal-9 and p-ERK inhibitor exhibited decreased expression of T-bet and IFN-γ, and increased expression of p-CREB and IL-10. These results suggest that the *T. gondii*-induced reduction of Gal-9 in dMφ promoted ERK phosphorylation by binding to Tim-3 on dNK, which in turn induced the upregulation of IFN-γ and downregulation of IL-10, thereby affecting the function of dNK.

## Conclusions

**Fig. 5:**
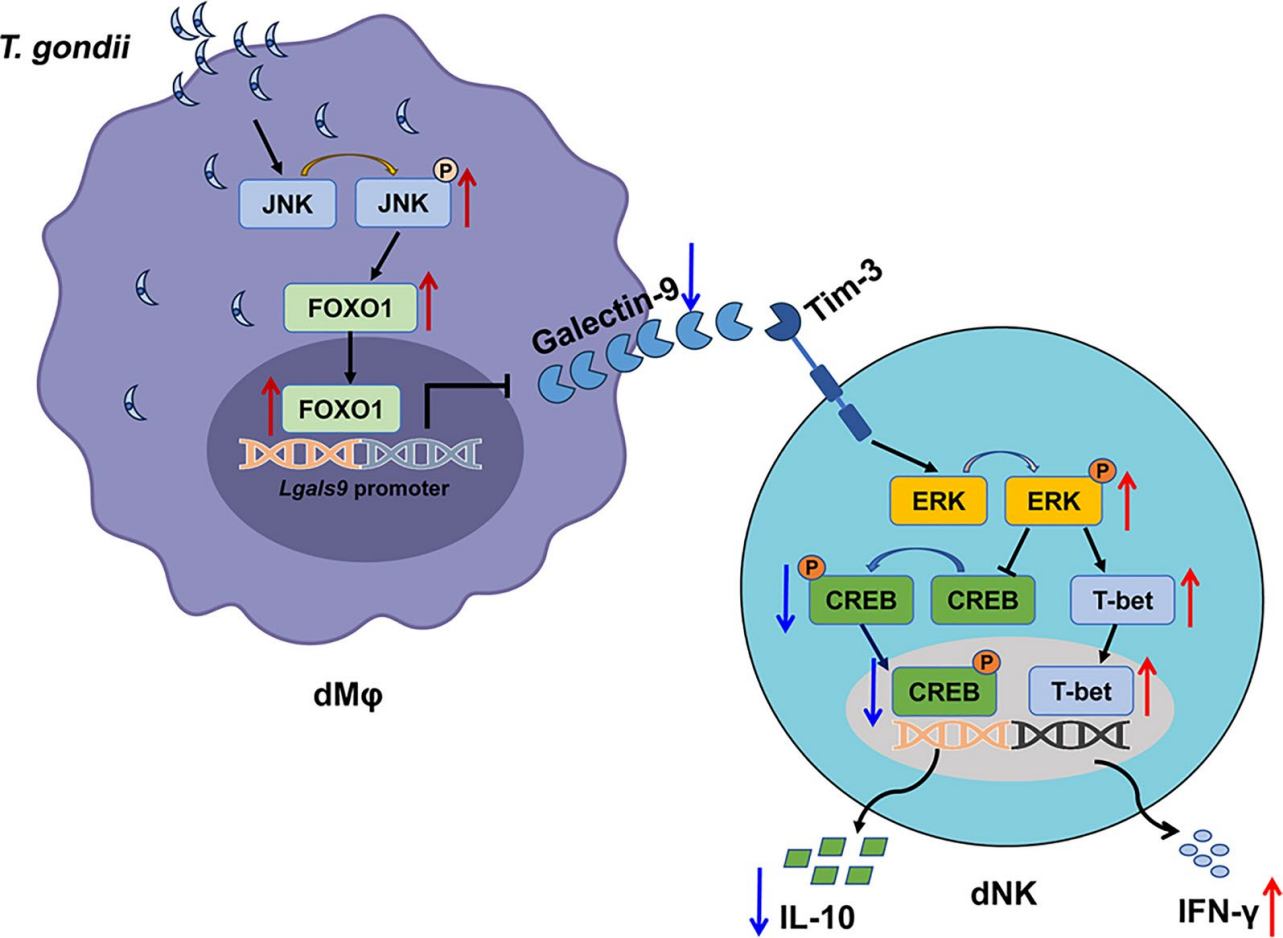
Schematic diagram illustrating the molecular mechanism whereby changes in Gal-9 expression levels in dMφ after *T. gondii* infection affect the maternal–fetal tolerance function of dNK cells. *T. gondii* infection could suppress Gal-9 expression in dMφ by activating the JNK/FOXO1 signaling pathway. The reduction of Gal-9 in dMφ further increases the phosphorylation of ERK in dNK cells via Gal-9/Tim-3 interaction, which resulted in elevating IFN-γ level and decreasing IL-10 level, and affected function of dNK cells

In summary, *T. gondii* infection suppressed Gal-9 expression in dMφ by activating the JNK/FOXO1 signaling pathway, and reduction of Gal-9 resulted in elevation of IFN-γ and suppression of IL-10 in dNK cells, thereby affecting dNK cell function and disrupting maternal–fetal homeostasis, ultimately resulting in adverse pregnancy (Fig. 5). This study provides new insights for the molecular mechanisms of the adverse pregnancy outcomes caused by *T. gondii*.

### Supplementary Information

Additional file 1: Table S1. The primers used for ChIP-qPCR.

Additional file 2: Table S2. Reagents and antibodies used in this study.

## Data Availability

All data generated in this study are presented within the published article.

## Abbreviations

dMφ: Decidual macrophage
dNK: Decidual natural killer cell
Tim-3: T-cell immunoglobulin domain and mucin domain-containing protein 3
FBS: Fetal bovine serum
FOXO1: Forkhead box protein O1
JNK: c-Jun N-terminal kinase
ERK: Extracellular signal-regulated kinase
CREB: Cyclic AMP (cAMP)-response element-binding protein
T-bet: T-box expressed in T cell
IL-10: Interleukin 10
IFN-γ: Interferon gamma
