# Supplementary material for: The effect of Toxoplasma gondii infection on galectin-9 expression in decidual macrophages contributing to dysfunction of decidual NK cells during pregnancy
Source: Parasit Vectors. 2024 Jul 10;17:299. doi: 10.1186/s13071-024-06379-2 (PMC11234737; doi:10.1186/s13071-024-06379-2)
Supplement: Supplementary file 1 — Additional file 1: Table S1. The primers used for ChIP-qPCR. [file 13071_2024_6379_MOESM1_ESM.docx]

Table S1: The primers used for ChIP-qPCR

| Domain of *Lgals9* promoter |  | Primers |
| --- | --- | --- |
| -1185 to -1171 bp, D1 |  | Fs: TGGATCTGGCTTCTTCTGCTC  Rs: GCTTTACTCATCATTGCCCC |
|  |  |  |
| -1009 to -1022 bp, D2 |  | Fs: TGAAATTGTCCCTGGTGTCC  Rs: ATACGTGAGTCTGCACCTCT |
|  |  |  |
| -574 to -561 bp, D3 |  | Fs: GGCACCCCATACTGTAGAAT  Rs: ACATACAGAGAAGCTGAGGC |
|  |  |  |
| -141 to -128 bp, D4 |  | Fs: AGCATGCTTAGGACACACAG  Rs: TCCTTTGTAGAGGGAACGAC |
|  |  |  |
