# Supplementary material for: The effect of Toxoplasma gondii infection on galectin-9 expression in decidual macrophages contributing to dysfunction of decidual NK cells during pregnancy
Source: Parasit Vectors. 2024 Jul 10;17:299. doi: 10.1186/s13071-024-06379-2 (PMC11234737; doi:10.1186/s13071-024-06379-2)
Supplement: Supplementary file 2 — Additional file 2: Table S2. Reagents and antibodies used in this study. [file 13071_2024_6379_MOESM2_ESM.docx]

Table S2: Reagents and antibodies used in this study

| Reagents and antibodys |  | Company |
| --- | --- | --- |
| PE-Cy7 anti-human CD14 |  | Biolegend, USA |
| PE-Cy7 anti-mouse F4/80 |  | Biolegend, USA |
| APC anti-human Galectin-9 |  | Biolegend, USA |
| APC anti-mouse Galectin-9 |  | Biolegend, USA |
| PE anti-human CD209 |  | Biolegend, USA |
| APC anti-human CD206 |  | Biolegend, USA |
| anti-Galectin-9 antibody |  | Proteintech, CHN |
| anti-FOXO1 antibody |  | Proteintech, CHN |
| anti-JNK antibody |  | Proteintech, CHN |
| anti-p-JNK antibody |  | Proteintech, CHN |
| anti-ERK antibody |  | Proteintech, CHN |
| anti-p-ERK antibody |  | Proteintech, CHN |
| anti-CREB antibody |  | Proteintech, CHN |
| anti-p-CREB antibody |  | Proteintech, CHN |
| anti-IL10 antibody |  | Wanleibio, CHN |
| anti-IFN-γ antibody |  | Bioss, CHN |
| anti-T-bet antibody |  | Wanleibio, CHN |
| Goat Anti-Rabbit IgG H&L (HRP) |  | Proteintech, CHN |
| p-ERK inhibitor (PD98059) |  | MCE, USA |
| Foxo1 inhibitor(AS1842856) |  | MCE, USA |
| p-JNK inhibitor(SP600125) |  | MCE, USA |
| recombinant human Gal-9 protein |  | MCE, USA |
| Tim-3-neutralized antibody |  | eBioscience, USA |
| EasySep^TM^ Human CD14 Positive Selection Kit II |  | Stemcell, CA |
|  |  |  |
| Reagents and antibodys |  | Company |
| EasySep^TM^ Human NK Cell Isolation Kit |  | Stemcell, CA |
| jetPRIME® transfection Reagent |  | Biosource, CHN |
| Pierce Magnetic ChIP Kit |  | ThermoFisher, USA |
| RPMI 1640 |  | Meilunbio, CHN |
| PCDNA3.1-FOXO1 (human)-3xHA-SV40-Neo |  | Miaoling Biology, CHN |
